# Telemedicine as an effective intervention to improve antibiotic appropriateness prescription and to reduce costs in pediatrics

**DOI:** 10.1186/s13052-017-0423-3

**Published:** 2017-11-17

**Authors:** Jacopo Ceradini, Alberto Eugenio Tozzi, Patrizia D’Argenio, Paola Bernaschi, Lucia Manuri, Carla Brusco, Massimiliano Raponi

**Affiliations:** Bambino Gesù Children’s Hospital IRCCS, piazza Sant’Onofrio 4, 00165 Rome, Italy

**Keywords:** Telemedicine, Antibiotics, Antibiotics stewardship program, Hospital infection

## Abstract

Implementation of antimicrobial stewardship program is a pivotal practice element for healthcare institution. We developed a remote infectious disease consultancy program via telemedicine in a high-specialized pediatric cardiac hospital. A consultation for antibiotic strategy for each patient was available via telemedicine in addition to biweekly discussion of all clinical cases. Aim of this study was to evaluate the impact of the remote stewardship program in terms of a) appropriateness of antibiotic prescription; b) incidence of multi-resistant infection; and c) cost. A ‘before - after’ study was performed comparing the period immediately before starting the program and one year after. There was a trend in the reduction of nosocomial infectious disease rate (9.5 vs 6.5 per 1000 person days), with a reduction in the overall antibiotic cost (25,000 vs 15,000 EUR) and in the average antibiotics packages used per admission (9 vs 6.7 packages). A significant reduction in the multi-drug resistant isolation rate was observed (104 vs 79 per 1000 person days, *p* = 0.01). In conclusion, the infectious disease meeting via telemedicine has been an effective tool for economic and professional development and multidisciplinary management of complex patients. The appropriate use of antibiotics reduced the multi-drug resistant bacteria selection, thus improving patient safety.

## Introduction and background

Improving the use of antibiotics is a key strategy to address antibiotic resistance and an important issue for patient safety and public health [1]. Antibiotic Stewardship Programs (ASP) are hospital-based programs dedicated to improving antibiotic use, with the aim to increase the appropriateness of prescriptions, to optimize the infection treatments and to minimize adverse events associated with antibiotics use [2, 3]. The Centers for Disease Control and Prevention (CDC) recommends that all acute care hospitals implement ASP in recognition of the urgent need to improve the use of antibiotics in hospitals and the benefits of ASP [4]. The implementation of these programs is generally achieved through educational activities that require intensive on-site activities and may be more difficult in suburban hospitals [5]. On June the 11th, 2010, a new pediatric cardiology clinical center, Centro Cardiologico Pediatrico del Mediterraneo” (**CCPM**), was founded in Sicily following an agreement between the public health agency of Sicily region, and The Bambino Gesù Children’s Hospital (**OPBG**), the largest pediatric research hospital within the Italian Healthcare System. The CCPM, which is part of Taormina hospital (220 beds), includes an intensive care unit, an ordinary ward, a cardiac outpatient center, and an operating room with a Hybrid Cath lab. The center performs complex treatments like sternotomies, interventions with extracorporeal circulation, cardiac catheterizations, ExtraCorporeal Membrane Oxygenation (ECMO) procedures, mechanical ventilation, on children often in critical conditions and low birth-weight requiring hospital-wide support for long periods. Despite an active surveillance system of nosocomial infections in ICU (Intensive Care Unit) by the CCPM health personnel, nor an infectious disease expert was available onsite, neither molecular biology tools were available in the laboratory. In January 2015, after a cluster of pneumonia patients admitted in ICU, OPBG decided to start a remote antimicrobial stewardship program for CCPM with a multidisciplinary team using telemedicine.

The aim of this study is to evaluate the impact of the remote stewardship program compared to the previous period in terms of: a) appropriateness of antibiotic prescription; b) incidence of multi-resistant infections; and c) costs.

## Methods

The remote stewardship program consisted of a biweekly discussion of all clinical cases admitted in CCPM and a review of antibiotic strategies for each patient. Telepresence techniques were used with commercial tools (total 25 meetings in a year) integrated with a high-definition camera, microphones, real-time sharing of files, desktop sharing and sharing of the cardiac radiology information system. During the online meetings, cardiologists, anesthesiologists, and surgeons from CCPM met infectious diseases and microbiology specialists from OPBG. Clinical records, diagnostic images, and other relevant reports were shared and reviewed. Antibiotic strategies were agreed and evaluated according to the discussion. Extra meetings were held in case of need. A teleconsultation tool, allowing consultation between a provider and specialist at distance using either store and forward telemedicine or real time videoconferencing, has been used only in selected cases.

In order to measure the intervention impact, we performed a ‘before - after’ study comparing a period immediately before the intervention (1 January 2014–1 March 2015) to a post-intervention period (1 March 2015–1 March 2016).

We assessed the following measures: a) incidence of nosocomial infections; b) incidence of isolation of multi-drug resistant bacteria; c) direct and indirect costs including those associated with antibiotic treatments and length of stay. The personnel involved was interviewed concerning their satisfaction in using teleconsultation for antibiotic stewardship.

Continuous variables are reported as the mean ± SD and data ranges or median and 1st-3rd quartile, as appropriate. Differences between groups were analyzed with Student’s t test for normally distributed variables or with the Wilcoxon test. Rates were compared through the mid-p test. Statistical significance was considered for *p* < 0.05. Analyses were performed through STATA software.

## Results

In the pre-intervention period 683 patients were included versus 531 patients in the post-intervention, as shown by the average weight Diagnostic Related Group (DRG)'s, populations are comparable and characterized by high complexity care (Table 1).

**Table 1:** Sample characteristics before and after intervention

Characteristic	Pre	Post	Significance
Admissions	683	531	
Average hospital stay, days	8.4 ± 11.9	8.4 ± 11.7	–
Average ICU stay, days	6.2 ± 10.8	6.1 ± 12.9	*p* = 0.92
Median age, years	1.7 (0.2–7.4)	2.1 (0.2–9.5)	*P* = 0.42
Average weight DRG	2.3	2	
ICU infection rate per 1000 days people	9.5	6.05	*p* = 0.23
MDR isolation rate	104	79	*P* = 0.01

Data are mean ± SD and median (1st and 3rd quartile) as appropriated. *ICU* Intensive Care Unit, *MDR* Multi-Drug Resistant

The main topics discussed during the meetings were monitoring and managing of Multi-Drug Resistant (MDR) Enterobacteriaceae and the antibiotic prophylaxis protocols in surgery. Furthermore, management of diagnostic specimens submitting to the microbiology laboratory was modified. To improve accuracy of microbiological diagnosis, CCPM clinicians ensured that diagnostic specimens were properly obtained and promptly submitted to the microbiology laboratory, preferably before the institution of antimicrobial therapy. In fact, before ASP meetings, a major issue was the timing of initial (empirical) therapy that should be guided by the urgency of the situation and the clinical presentation. The meetings trained clinicians about: 1) understanding the difference between empirical and definitive therapy; 2) identifying opportunities to switch to narrow-spectrum, more effective agents for the shortest duration necessary; 3) understanding drug characteristics that are peculiar to antimicrobial agents (such as pharmacodynamics and efficacy at the site of infection) and 4) understanding antimicrobial susceptibility testing.

The average stay in pediatric ICU was not significantly different between the pre and post ASP meetings (6.2 ± 10.82 vs 6.1 ± 12.91 days, *p* = 0.92).

The hospital infections in ICU rate per 1000/person days decreased from 9.5 in the pre-intervention period to 6.1 for 1000/person days in the post intervention, though the difference was not statistically significant (*p* = 0.23).

The rate of MDR isolation decreased from 104 to 79 per 1000 person days with a significant reduction of 25% (*p* = 0.01). We are aware that other variables as seasonal variation or surgical case load during that time have not been considered in this analysis, but we assumed that the reduction in the antibiotics consumption is the first factor associated to the reduction of the MDR rate.

The overall costs of antimicrobials fell dramatically, from 25,000 EUR in 2014 to 15,000 EUR. The mean cost of antibiotic therapy per admission was 43 EUR vs 27 EUR. The reduction was justified by the lower utilization of complex molecules that were used only in selected cases after teleconsultation with the specialist in infectious diseases. Consumption of systemic antimicrobials (ATC J01) was 6126 packs in 2013; 5296 in 2014; and 3779 in 2015 (the difference between 2013 and 2015 is 2347 drug packages), with a mean packages consumption of 9 vs 6.7 in 2014 and 2015, respectively.

The medical doctors interviewed were satisfied by the intervention and reported that they had improved their knowledge about the antibiotics prescription and the protocols adopted.

## Discussion

In this scenario where the health service has to promote cost reduction, network organization and clinical specialization, the information technology (ITC) offers economic sustainable tools to support professional training and patient safety. Telemedicine technologies are rapidly being integrated into infectious diseases programs with the aim of increasing access to infectious diseases specialty care and reducing costs [6]. In France, some telemedicine experiences have been used to train doctors and supervising young doctors in the management of infectious diseases in medium-sized hospitals [7]. Antimicrobial stewardship through telemedicine has been performed successfully in a community hospital in South America [8]. The experience of the CCPM shows how the second opinion of an expert in infectious diseases may change the antibiotic policy. This experience with telemedicine might also be extended to other disciplines such as radiology and neonatology, creating the conditions for a permanent training of doctors and decision-support activities in the most difficult cases. A key telemedicine advantage is the increased access to specialized hospital facilities for suburban hospitals or isolated areas. Telemedicine is not just a simple conference call, it is a more complex tool where different imaging modalities are melted together and experts from several specialties discuss evaluating at the same time the clinical and individual data of the patient but also the epidemiologic factors. To resolve complex problems it is fundamental the interaction between different experts which include not only verbal communication but also non-verbal communication, as eye-contact.

## Conclusion

The infectious diseases meeting at the CCPM was an effective tool for economic and professional development, and multidisciplinary management of complex patients. It allowed the sharing of protocols and best practices, promoted the prudent use of antibiotics [9] and it was also associated with direct economic savings.

The appropriate use of antibiotics reduces the selection of MDR bacteria, and the risk for patients and health workers [10], although these indirect costs were not measured.

## Abbreviations

AB: Antibiotic
ASP: Antimicrobial stewardship program
CCPM: Centro Cardiologico Pediatrico del Mediterraneo
DRG: Diagnostic Related Group
ICU: Intensive Care Unit
MDR: Multi-Drug Resistant
OPBG: Bambino Gesu’ Pediatric Hospital
